# Hybrid surgery versus anterior cervical discectomy and fusion in multilevel cervical disc diseases: a meta-analysis: Retraction

**DOI:** 10.1097/MD.0000000000019078

**Published:** 2020-01-31

The article, “Hybrid Surgery Versus Anterior Cervical Discectomy and Fusion in Multilevel Cervical Disc Diseases: A Meta-Analysis”,^[1]^ which appears in Volume 95, Issue 21 of Medicine, was incorrectly retracted in October 2019^[2]^ due to its similar title to a published article in Volume 5 of *Scientific Reports*.^[3]^ The retracted article has been republished in Vol 99, Issue 5 ^[4]^ of *Medicine* under the new title “Comprehensive Analysis of Hybrid Surgery and Anterior Cervical Discectomy and Fusion in Cervical Diseases: A Meta-Analysis”. No other changes have been made to the republished article aside from the title.
